# Risk Haplotype of BTNL2 Predisposes Male Patients to NSTEMI: A Genetic and Functional Study

**DOI:** 10.21203/rs.3.rs-9200701/v1

**Published:** 2026-03-25

**Authors:** A. Inkeri Lokki, Juha Sinisalo, Kitty Aierken, Marita Kalaoja, Mitja Lääperi, Jessica Koski, Veikko Salomaa, Markus Perola, Johannes Kettunen, Markku Varjosalo, Hanna Jarva, Pirkko Pussinen, Sarah Klein, Katariina Öörni, Mikko Mäyränpää, Steven Mack, Marja-Liisa Lokki

**Affiliations:** University of Helsinki; Helsinki University Hospital and University of Helsinki; Helsinki University Hospital; Biocenter Oulu, University of Oulu; Faculty of Medicine, Physiology, University of Helsinki; University of Helsinki; Department of Public Health and Welfare, Finnish Institute for Health and Welfare; Finnish Institute for Health and Welfare; University of Oulu; Institute of Biotechnology, Helsinki Institute of Life Science HiLIFE, P.O Box 56, 00014 University of Helsinki, Helsinki, Finland; Helsinki university hospital; University of Helsinki; Cell Signaling Technology; Wihuri Research Institute; Helsinki University Hospital; University of California, San Francisco; Helsinki University

## Abstract

Immunogenetic factors constitute major pathways contributing to the susceptibility to atherosclerosis and coronary artery disease. Through targeted whole-genome sequencing and replications in sex-specific cohorts with ST-elevation myocardial infarction (STEMI) or non-ST-elevation myocardial infarction (NSTEMI) we discovered a novel haplotype in the butyrophilin-like 2 (BTNL2) gene that predisposes men to NSTEMI. This risk haplotype is associated with the number and composition of extra-large high-density lipoproteins, and enhanced aggregation of low-density lipoproteins. Affected individuals have changes in BTNL2 expression. Furthermore, patients with risk haplotype and decreased BTNL2 serum concentration had improved survival. Our results identify BTNL2 as a compelling candidate gene in men with NSTEMI and suggest a previously unrecognized immuno-lipid regulatory mechanism contributing to disease susceptibility and outcome. The study highlights the importance of precise clinical characterisation and specific diagnoses in genetic studies of cardiovascular diseases.

## Introduction

Cardiovascular diseases are the leading cause of death in the Western world, the majority of deaths are due to coronary artery disease (CAD)^1^. Single nucleotide polymorphisms (SNP, 4–5 million SNPs/genome) account for less than 0.1 percent of the human genome sequence but explain over 20% of the heritability of CAD ^2^. After analysing millions of genetic markers for association with CAD, there are today more than 300 CAD loci suggested by numerous studies (reviewed by ^3^). Genetic variation is expected to affect susceptibility to CAD either by a) regulatory elements affecting transcriptional activity e.g. amount of RNA or protein produced, or b) coding variation affecting binding or signalling properties of the molecules. While the individual risk variants are located primarily at the regulatory regions and have modest effect sizes (reviewed by ^4^) their effect can be substantial on disease risk. In contrast, only a few studies exploring exome sequenced CAD materials have been published ^5,6^. A large-scale genome wide risk score (metaGRS) comprising 1.7 million variants revealed that its predictive ability was largely independent of established traditional risk factors, indicating that genetic information complements conventional risk assessment ^7^.

SNPs with exceptionally large effect are seen with variants at the major histocompatibility complex region (MHC 6p21.31), where genes encode proteins that regulate inflammation, immunity, and defensive mechanisms against microbes and altered host proteins. The MHC is a highly polymorphic, gene-dense region spanning nearly 4 Mb with complex allelic and genetic architecture, presenting challenges for SNP analysis ^8^. Several SNPs in the MHC region have been associated with CAD ^9–13^. In our previous study the haplotype containing butyrophilin-like 2 (*BTNL2)* and *HLA-DRA* SNPs and HLA-DRB1*01 gene associated with myocardial infarction (MI) in three Finnish case-control populations ^14^.

BTNL2 is a single-pass type II membrane protein consisting of 455 amino acids. BTNL2 is related to the B7 family of immunoregulatory proteins, whose function is poorly characterized. The B7 regulators have known associations to polygenic traits, including CAD ^14,15^. While butyrophilin (Btn1a1) has a role in lipid secretion to milk during lactation, BTNL2 is known to act as a negative costimulatory receptor with a regulatory role in T-cell homeostasis. The gene coding for BTNL2 is under a stringent selection pressure making *BTNL2* an attractive target for candidate gene studies in complex inflammatory diseases ^16^*. BTNL2* consists of seven exons and several splicing variants of the gene product have been described ^17^.

In this study, we deep-sequenced the *BTNL2* gene, including the flanking upstream and downstream regions to examine if the *BTNL2* gene has wider and HLA-independent roles in increasing the risk for myocardial infarction (MI). We replicated our suggestive result in a prospective cohort and show that variants in the *BTNL2* are associated with CAD susceptibility, protein expression, lipid metabolism and survival.

## Methods

### Study cohorts

Patient samples from Corogene-cohort (a prospective cohort of patients undergoing coronary angiogram for genetic studies) and Oxi-trial (a cohort of myocardial infarction patients for placebo-controlled hydroxychloroquine trial), and control samples from FINRISK, a Finnish national population-based health examination survey cohorts from years 1992, 1997, 2002 and 2007 were analysed ^18–20^. A detailed description of the study cohorts is available in the supplementary methods. All studies were conducted according to the principles of the Declaration of Helsinki. The subjects gave their informed consent and ethics committees of the participating study centres have approved the research protocols (Dnro 205/E0/2007 and Dnro 29/13/03/01/2011 as an amendment for Corogene, Dnro 148/13/03/01/2015 for OXI-cohort and Dnro 38/96, Dnro 558/E3/2001 and the amendment 229/E0/2006 for FINRISK).

In the discovery study we sequenced *BTNL2* for 24 acute coronary syndrome (ACS) patients of the Corogene cohort and for 23 healthy control samples selected from the FINRISK study participants. Patients and controls were from the same geographic region, and were genotype matched: one third of both groups were homozygous, heterozygous or negative to previously described *BTNL2* SNPs known to associate to ACS^14^

In the first replication, MI patients (n=1901) of the Corogene cohort and FINRISK controls (n=1354) were compared, of which 1024 had the NSTEMI diagnosis. For case and control characteristics, see **Table 1**. In a second replication, we analysed an additional subset of patients from the Corogene cohort (chronic CAD n=1606 and patients verified with angiography not to have CAD [non-CAD] n=1195) to replicate initial finding, see **Table 1**. For the mRNA studies, we used patients from the OXI cohort due to availability of suitable samples in this cohort.

### Genotyping methods

Detailed protocols are provided in the supplementary materials. Here follows a brief description of methods used.

#### Discovery study

The SeqTarget system (www.qiagenbioinformatics.com/) *was used for BTNL2 next-generation sequencing. Samples were purified and normalized according to the manufacturer’s instructions and analyzed on LabChipR GX* (www.perkinelmer.com.cn).

#### Replication studies

Selected SNPs were genotyped with the Sequenom platform (iPlex MassARRAY, San Diego, CA) at the Institute for Molecular Medicine Finland (FIMM, Helsinki, Finland). The quality control applied to the samples used Illuminus software from Illumina. Quality control checks required removal of individuals with (1) failed sex check, (2) low genotyping frequency (<95%), (3) excess heterozygosity, (4) non-European background based on multidimensional scaling, or (5) excess relatedness (PI-HAT>0.1). SNPs with a low call rate (<95%), low minor allele frequency, or low Hardy–Weinberg disequilibrium *P* value (<1×10^−6^) were excluded.

#### Genotyping of the OXI cohort

We extracted the DNA from whole blood and Sanger sequenced with forward primer 5’-TGCCTTCTGAATATCCACTGAAA-3’ and reverse primer 5’-TGTGTGACTTTGTCAGCTCAT-3’ the samples from the OXI cohort. Exact protocol is available at supplementary methods.

### BTNL2 expression studies

The MI patient cohort for *BTNL2* mRNA quantification studies is described in the supplementary methods. Different patient cohort was used due to availability of suitable samples in the OXI cohort. The mRNA was extracted, cDNA library was created, and RT-PCR quantification was done according to specifications in the supplementary methods.

#### BTNL2 protein analysis

To study, whether the *BTNL2* SNPs with strongest association identified by sequencing influence the molecular size of BTNL2 protein, we performed a Western blot analysis of patient sample aliquots of peripheral blood buffy coat cell lysates (white blood cells and platelets). We ran the samples into a 4–12% SDS-PAGE gel (Invitrogen, Waltham, MA, USA) under reducing conditions. The samples were then transferred to a nitrocellulose membrane and unspecific binding sites were blocked with 5% milk in 0.05%Tween/PBS for 1 hour. After blocking, the blots were incubated overnight with the BTNL2 antibody (55.3 μg/ml, Cell Signaling Technology, Danvers, MA, USA) in 1:40 milk/Tween/PBS at +4°C. The blots were washed with Tween/PBS and incubated with the secondary antibody HRP-goat-anti-rabbit (Jackson ImmunoResearch Inc, West Grove, PA, USA) in 1:10.000 milk/Tween/PBS. Electrochemiluminescence was used to visualize the bands.

To confirm that the bands in Western blot contained BTNL2 protein, we performed a mass spectrometry analysis. We ran the samples into an SDS-PAGE gel as described above. The gel was stained with GelCode Blue protein stain (Thermo Fisher Scientific, Waltham, MA, USA). The specific bands were identified by comparison with the Western blots and cut out of the gel and analysed by mass spectrometry using standard procedures ^21^. Human BTNL2 recombinant protein was used as a positive control (NovoPro, Shanghai, China).

#### BTNL2 concentration measurements

Serum BTNL2 concentrations were quantified by a commercial enzyme-linked immunosorbent assay (ELISA) according to the manufacturer’s instructions (Wuhan Fine Biotech Co, Wuhan, Hubei China). The concentration range of the standard curve was 0.312–20 ng/ml. We used undiluted patient serum samples and the inter-assay coefficient of variation was 9.2%.

#### Measurements of LDL aggregation

Variability in LDL aggregation-susceptibility between individuals was quantified by previously published methods ^22,23^. Briefly, LDL was isolated from plasma samples by ultracentrifugation, aggregation of each LDL preparation was induced *ex vivo* by incubation with human recombinant secretory sphingomyelinase (hrSMase) produced in house^23^, and the kinetics of aggregation were followed in real time by measuring the growth of the aggregates by dynamic light scattering using Wyatt DynaPro Plate Reader II (Wyatt Technology, USA). Aggregate size (nm) after incubation for 2 h was used as a measure of the aggregation susceptibility of LDL. The samples were analysed blinded.

#### Metabolome measurements

The metabolic measures were quantified with a high-throughput nuclear magnetic resonance spectroscopy measure and an in-house developed quantification software (Nightingale Health, Helsinki, Finland). The metabolomics platform quantifies from baseline serum samples 228 metabolic measures representing multiple metabolic pathways, including amino acids, glycolysis-related metabolites, fatty acids and detailed lipoprotein lipid profiles, covering triacylglycerol, total cholesterol, non-esterified cholesterol, esterified cholesterol and phospholipids within 14 subclasses ^24^.

### Statistical analyses

#### *BTNL2* sequence analysis

The BIGDAWG R package was applied to calculate p-values for overall (kx2) test of heterogeneity, and to calculate odds ratios (ORs), 95% confidence intervals (CIs) and p-values in individual (2×2) chi-squared association tests, in case-control analyses^25^. Both individual SNPs and SNP haplotypes (generated via BIGDAWG’s implementation of the expectation-maximization [EM] algorithm) were tested, in two sets of evaluations; (1) excluding all subjects with any missing data, and (2) allowing up to two missing (unknown) variants per subject and excluding any subjects with more than two unknown variants. Altogether 525 SNPs were studied.

#### Statistical analyses

Association analyses for allelic data were calculated with Chi-square tests with the Yates value correction for continuity. Significances of differences between measurements and *BTNL2* alleles were analysed by the Mann-Whitney U test and the Chi-square test.

We fitted logistic regression models comparing the effect of the genotype between CAD and non-CAD for both men and women along with NSTEMI, and STEMI against controls in men. The models were adjusted for age and age squared. The 2-sided *P* value for statistical significance was *P*<0.05, and the Benjamini-Hochberg procedure ^26^ was used for correcting for multiple testing. Analyses were executed with the IBM SPSS Statistics 20 Statistical Package for the Social Sciences, R software version 4.5.1 ^27^, GraphPad Prism 8 for Windows (GraphPad Software, San Diego, CA, USA), and gPLINK software (version 2.050)^28^ or PLINK software (version 1.07)^29^.

Associations between *BTNL2* risk haplotype and metabolic measures were assessed using linear regression. Metabolic measures were scaled to standard deviation (SD) units to allow comparison across multiple measures. Scaled concentration of each metabolic measure was used as an outcome variable and *BTNL2* haplotype status and case-control status as explanatory variables in the model. An interaction term between *BTNL2* haplotype status and case-control status was added to evaluate possible differences in the effect of *BTNL2* status between cases and controls. Analyses were adjusted for age and sex. Individuals homozygous for the *BTNL2* risk haplotype were identified as having two risk alleles in each of the nine SNPs. 44 principal components were identified to account for 99% of the variation in the data. To correct for multiple testing P < 0.0011 (0.05/44) was taken as evidence of association. Analyses were done in R version 3.5.3.

#### Linkage analysis

Linkage disequilibrium (LD) analysis was conducted with LD Software (www.ldlink.nci.nih.gov) or PLINK software (version 1.07).

## Results

### *BTNL2* associates with coronary artery disease

In the initial study, we sequenced the *BTNL2* gene and flanking upstream and downstream regions (32.7 kb) from 24 MI patients and 23 healthy controls. Sequencing revealed 525 SNPs in total. A novel *BTNL2* haplotype T~C~G~A~C~C~G~A~C~C~A~T~G~G of 14 SNPs (lead SNP rs17202358, rs62402759, rs62404560, rs113669229, rs4248166, rs4959097, tagging SNP rs2294884, rs2294883, rs2294882, rs2294881, rs112748105, rs3763304, rs3763306, rs4935352) was discovered to be highly predisposing (OR=5.79, 95% CI=2.05–17.24, unadjusted p=1.7 × 10^−4^) to MI. The lead SNP resides in the structures of three nearby genes, in the downstream region of *BTNL2*, at the end of intron 2 of the ncRNA gene *HCG23* (HLA Complex Group 23) and in the antisense RNA1 of *BTNL2* and *TSBP-1* (Testis Expressed Basic Protein 1). The rest of the 13 variants are in linkage with allelic frequency differences with the lead SNP rs17202358 (R^2^=0.5661, D’=1.0). All other variants, in total linkage with each other (R2=1.0, D’=1.0), reside in *BTNL2* intron 4 except rs3763306 and rs4935352, which are in intron 3. Detailed information of the SNP associations is presented in Supplementary table 1.

In the first replication, nine SNPs of the *BTNL2* risk haplotype (rs62402759, rs4248166, rs4959097, rs2294884, rs2294883, rs2294881, rs3763304, rs3763306, rs4935352) were successfully analysed in 1901 MI patients with age, sex and geography-matched healthy controls (n=1354; **Table 2a**). The *BTNL2* SNPs associated significantly with MI (rs2294884, p=0.004, OR=1.17, 95% CI=1.05–1.30, adjusted p=0.015). When MI-patients were further analysed by gender and diagnosis-specific subgroups (ST-elevation MI [STEMI] or non-ST-elevation MI [NSTEMI]), association was found in NSTEMI men (n=695) *vs*. healthy control men (n=759) (rs2294884, p=1.84 × 10^−4^, OR=1.30, 95% CI=1.131–1.485, adjusted p=8.11 × 10^−4^) but not in women. The genetic model analysis reveals that in male NSTEMI patients, the association is driven by the dominant model of genotype association indicating, that a single copy of risk allele G is sufficient for increased disease risk (**Table 2b**).

In the second replication, five risk SNPs in intron 4 (rs62402759, rs4248166, rs2294884, rs2294883, rs2294881) and two in intron 3 (rs3763306, rs4935352) were further analysed in chronic CAD patients (n=1606) vs. patients, who were verified with angiogram not to have CAD (n=1195). The *BTNL2* SNPs significantly associated with patients diagnosed with chronic CAD (rs2294884, p=0.01, OR=1.16, 95% CI=1.036–1.297, adjusted p=0.033). Again, the association was found in men with chronic CAD (n= 1116) vs. non-CAD men (n= 596) (rs2294884, p=0.002, OR=1.27, 95% CI=1.091–1.474, adjusted p=0.007) but not in women (**Table 2a**).

**Figure 1:**
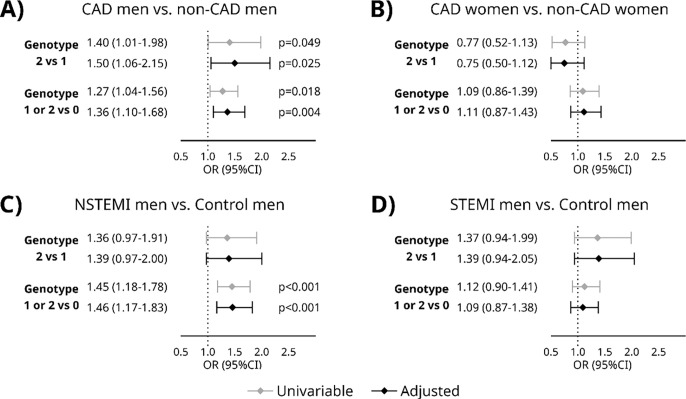
Logistic regression analyses for the genotype revealed significant association in (A) in the top panel, *BTNL2* risk haplotype homozygotes or heterozygotes in CAD males vs non-CAD males and in the bottom panel, homozygous CAD males vs non-CAD males. (B) *BTNL2* genotypes did not associate to disease risk in CAD women vs non-CAD women. (C) NSTEMI males vs control males for *BTNL2* risk haplotype homozygotes or heterozygotes. Significant association was discovered to disease risk in comparison of individuals without any risk alleles in comparison to those who had one or two disease alleles (bottom panel). No associations were detected in (D) STEMI men vs control men. Grey lines represent the univariable results, and the black lines represent results adjusted with age and age squared. NSTEMI – non ST-elevation myocardial infarction, CAD – coronary artery disease, nonCAD – no evidence of coronary artery disease OR (95%CI) – odds ratio (95% confidence interval)

In genotype analyses by logistic regression model, a significant association was detected for *BTNL2* tagging SNP risk homozygotes or heterozygotes in CAD men vs non-CAD men OR=1.27, 95% CI=1.04–1.56, p=0.018 and adjusted by age OR=1.36, 95% CI=1.10–1.68, p=0.004. In addition, among homozygous individuals, CAD men showed a significantly increased risk compared with non-CAD men after age adjusting OR=1.50 (95% CI=1.06–2.15, p=0.025). Furthermore, a significant association was observed between NSTEMI men and control men for *BTNL2* risk haplotype homozygotes or heterozygotes independently OR=1.45 (95% CI=1.18–1.78, p=4.3 × 10^−5^) and adjusted by age OR=1.46 (95% CI=1.17–1.83, p=0.001). Significant associations were not detected in CAD women vs non-CAD women or STEMI men vs control men (Figure 1).

### BTNL2: intracellular expression

**Figure 2:**
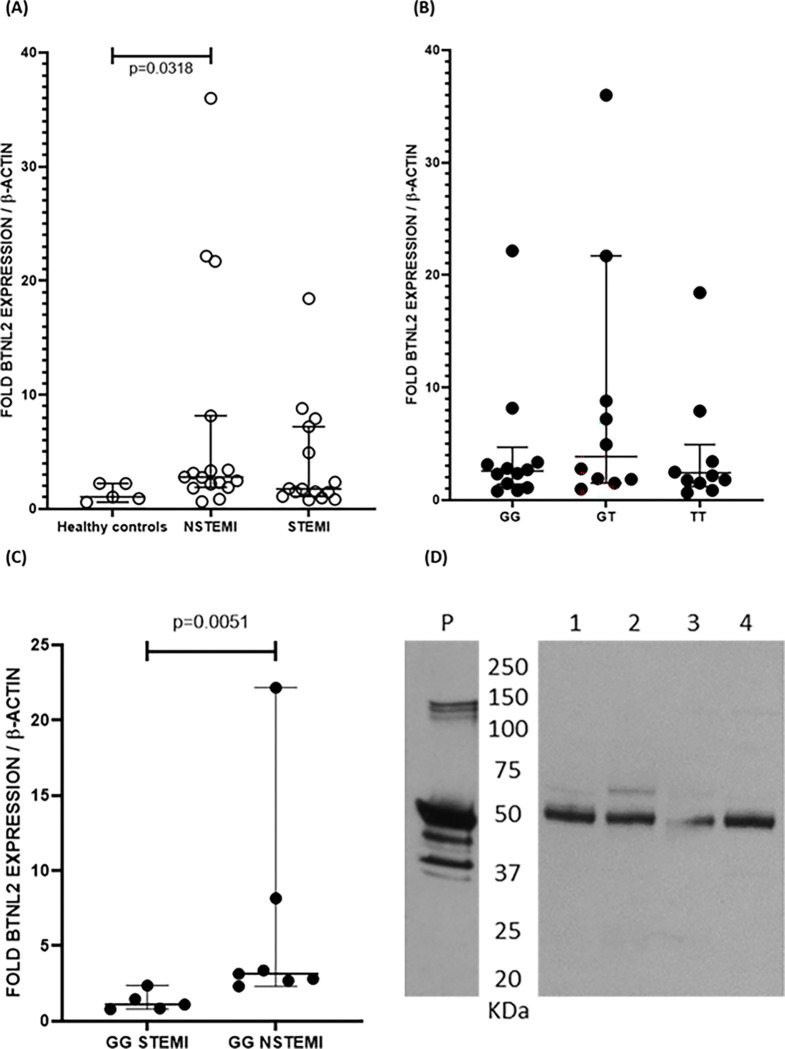
Expression of *BTNL2* mRNA in whole blood cells. (A) Myocardial infarction patients have an increased fold *BTNL2* expression (reference gene β-actin) in comparison to whole blood cells of healthy controls. (B) Among myocardial infarction patients, expression levels of *BTNL2* are not dependent on the *BTNL2* rs2294884 genotype. (C) Comparison of STEMI patients with GG and NSTEMI patients with GG genotype reveals that in peripheral mononuclear blood cells, the GG genotype is associated with increased expression BTNL2 in patients with NSTEMI. (D) In Western blot analysis we used cell lysates from four patients of which two were the non-carriers (1 and 2) and two the carriers (3 and 4) of the *BTNL2* risk haplotype. P indicates human BTNL2 recombinant protein as positive control ran on the same gel. All patients had a BTNL2 band of ~50kDa.

Intracellular *BTNL2* expression was quantified at the mRNA level using white blood cells. We found that in NSTEMI male patients, *BTNL2* mRNA levels were increased in comparison to healthy controls (p=0.032) (Figure 2a). However, we did not detect differences in mRNA levels between MI patients with homozygous (GG), heterozygous (GT) or negative (TT) risk genotype (Figure 2b). However, in GG NSTEMI men, the *BTNL2* mRNA levels were increased in comparison to GG STEMI men (p=0.005) (Figure 2c). Furthermore, the BTNL2 protein was detectable in WBC lysates for all genotypes with no observable differences in protein size (Figure 2D).

### BTNL2: extracellular expression

**Figure 3:**
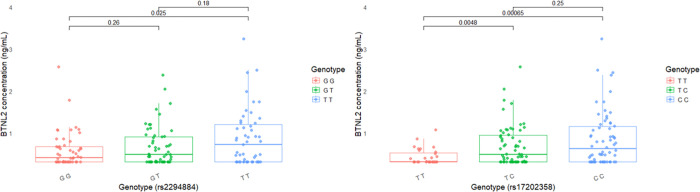
*BTNL2* variants effect the serum BTNL2 concentration in NSTEMI males’ sera. (A) The risk genotype GG in tagging SNP rs2294884 produces significantly less BTNL2 protein to serum than the wild type genotype TT. (B) For the haplotype lead SNP rs17202358 the differences are more pronounced. The heterozygotes and wild type samples have more serum BTNL2 when compared to the risk genotype TT. Three outlier samples were removed, which did not affect the statistical significance of the findings.

To quantify extracellular expression of BTNL2, we used ELISA to detect concentrations of soluble BTNL2 in sera of NSTEMI male patients. In the patients with the homozygous risk genotype GG in tagging SNP rs2294884, soluble BTNL2 was observed in lower concentrations in comparison to the patients without risk alleles (p=0.021, Figure 3a). The difference in serum soluble BTNL2 concentration was even more pronounced when the same patients’ concentrations were compared against risk genotypes detected by the lead SNP of the risk haplotype (rs17202358_T, Figure 3b). Here a statistical difference was observed not only between patients who are homozygous or negative for the risk haplotype (TT vs CC, p=0.001) but also between patients who are homozygous vs heterozygous for the risk haplotype (TT vs TC, p=0.001).

### *BTNL2* risk genotype associates with lipid metabolites related to MI

We analysed 228 metabolites related to lipid biosynthesis, amino acids, glycolysis, and lipoprotein subclasses to study the putative roles of SNPs from *BTNL2* risk haplotype and their effect on metabolism in 1232 Corogene patients and 551 FINRISK controls (**Table 3**).

**Figure 4:**
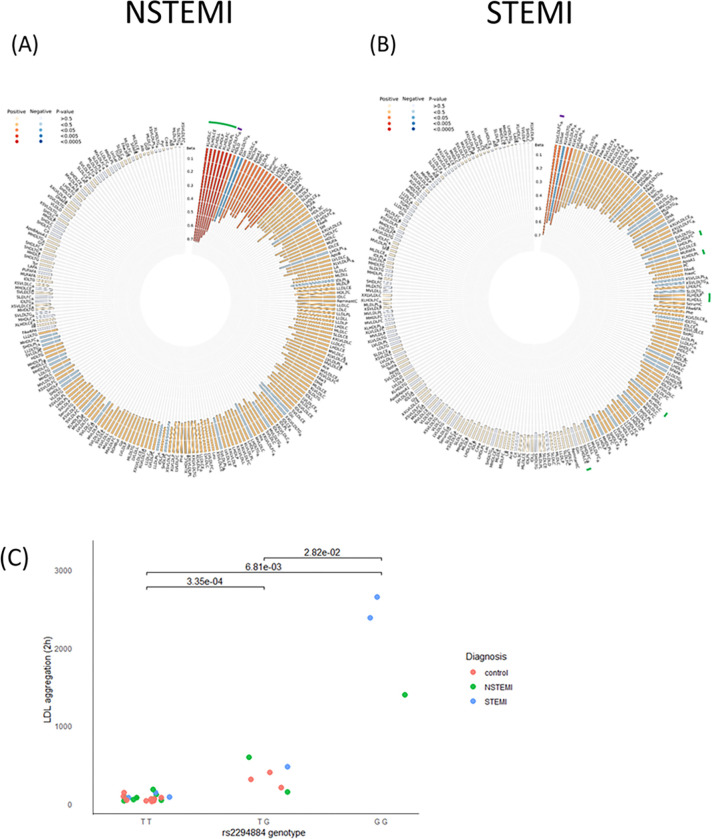
The risk genotype GG for tagging variant rs2294884 is associated with changes of metabolic measures. (A) In male patients with NSTEMI myocardial infarction, the metabolites with significant changes are indicated in green (listed in table 3). The colour and depth of the metabolite specific indicator towards the center of the circus plot reflects the direction (red – increase, blue – decrease), significance (dark – more, light – less significant) and magnitude (depth – beta) of the change. (B) In male patients with STEMI myocardial infarction, the metabolites related to extra-large HDL particles and their composition are not significant (green indicators) but instead, the free cholesterol to total lipids ratio in very large VLDL is significantly increased, indicated by purple (also indicated in figure A, where not statistically significant). (C) The risk genotype GG results in increased LDL aggregation The sizes of LDL aggregates after incubation for 2 h with hrSMase are shown. P-values displayed for each comparison.

Among the metabolic measures, the risk haplotype was associated with HDL and VLDL compositions. In male patients with NSTEMI, who were homozygous for the *BTNL2* risk haplotype (n=761) we found an increase in the amount of very large HDL particles (Beta=0.70, 95% CI=0.29–1.11, p=8 × 10^−4^). In these particles were enriched by total cholesterol, cholesterol esters and total lipids (Beta=0.7, 95% CI =0.30–1.10, p=7 × 10^−4^; Beta=0.7, 95% CI =0.30–1.11, p=7 × 10^−4^; Beta=0.70 95%, 95% CI =0.29–1.11, p=8 × 10^−4^, respectively; Figure 4a). These differences were associated with a significant interaction between genotype and disease status in homozygous male patients with NSTEMI. In male patients with STEMI, who were homozygous for the *BTNL2* risk haplotype (N=521), a decrease in the ratio of free cholesterol to total lipids in very large VLDL (Beta= −0.60, CI95= −0.87- −0.24, p=7 × 10^−4^; was found (Figure 4b). This association was independent from the disease status. In homozygous men with NSTEMI, the ratio of triglycerides to total lipids in very large VLDL was increased (Beta=0.53, 95% CI =0.22–0.84, p=7 × 10^−4^, n=616). In contrast, in both homozygous NSTEMI and STEMI men we observed a genotype-associated decrease of similar magnitude in ratio of total cholesterol and free cholesterol to total lipids in very large VLDL (Beta=−0.54, CI95=−0.85- −0.23, p=0.0006; Beta=−0.57, 95% CI =−0.88- −0.27, p=2 × 10^−4^, respectively). In individuals, who were homozygous for the *BTNL2* risk haplotype, lipid metabolite levels did not show significant differences between patients and controls when all disease subtypes and both genders were grouped together.

### *BTNL2* risk genotype affects LDL aggregation

Across all diagnoses, LDL aggregation after two hours increased progressively with the number of risk alleles: wild-type individuals (rs2294884_TT) exhibited the lowest aggregation, heterozygotes showed an intermediate level (p = 0.028), and homozygous carriers of the risk allele G displayed the highest aggregation (Figure 4c, rs2294884_TT vs GG, p = 0.007).

### NSTEMI men with low-expressing *BTNL2* genotype are at highest risk of death

**Figure 5:**
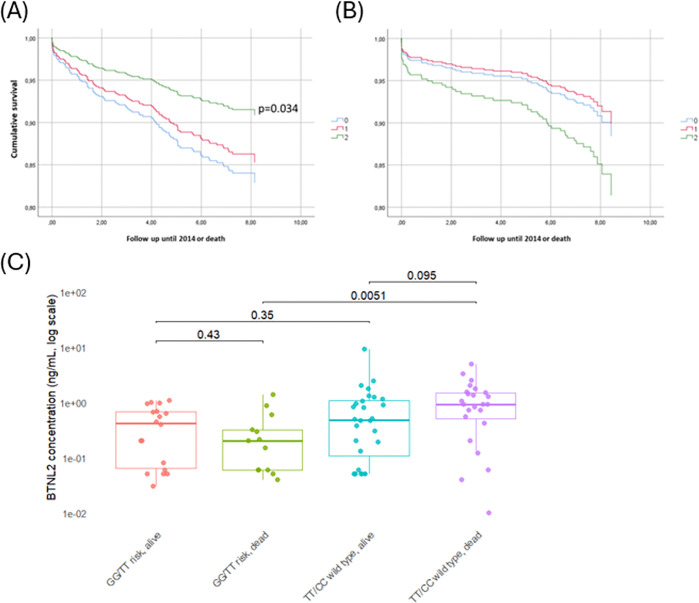
*BTNL2* affects the survival of MI patients. (A) The Kaplan-Meier survival plot shows that in male NSTEMI patients, the genotype associated with an increased risk of MI (GG) is associated with significantly better survival. (B) This association is not observed in male STEMI patients, instead, a trend of poor survival for GG patients is observed (not statistically significant). (C) Among NSTEMI male patients. a difference in genotype is detected in those that died during the nine-year follow-up time. Those patients who had the homozygous risk genotype GG in the tagging SNP rs2294884 and risk genotype TT in the lead SNP rs17202358, had significantly lower BTNL2 serum levels when compared to those dead patients, who did not have the risk haplotype. Values were winsorized to 95th percentile to make the plot cleaner, tests were done using the original data

NSTEMI male patients carrying the disease-associated genotype demonstrated improved survival over a nine-year follow-up period (p=0.034, Figure 5A) whereas male STEMI patients showed no association (Figure 5B). However, NSTEMI male patients, who died during follow-up and carried the homozygous risk genotypes (GG in the tagging SNP rs2294884 and TT in the lead SNP rs17202358), had significantly lower BTNL2 serum levels when compared with deceased patients without the risk haplotype (p=0.005, Figure 5C).

### Novel *BTNL2* risk SNPs are in LD with other *BTNL2* and flanking risk SNPs

The *BTNL2* risk SNP rs17202358 is in complete LD (R^2^=1.0, D’ =1.0) with the previously discovered ACS risk SNP for ^14^ (rs1555115 *BTNL2* downstream) whereas the remaining risk SNPs exhibited partial linkage (R^2^=0.566, D’=1.0). The *BTNL2* risk SNPs also showed relatively high LDs with two previously found risk SNPs (rs3763313; *BTNL2* promoter region and rs17496307; *HLA-DRA* promoter region). Furthermore, the risk SNP rs17202358 was in total LD (R^2^=1.0, D’ =1.0) with *BTNL2* missense variants p.Met380Ile and p.Pro379Leu. (Supplementary figure 1).

## Discussion

We identified a novel *BTNL2* haplotype consisting of fourteen SNPs predisposing to MI. One of these SNPs is in the *BTNL2* 3’ untranslated region, eleven are in intron 4, and two are in intron 3. This disease association was replicated in two prospective case-control studies confirming its link to MI and chronic CAD. Notably, a closer analysis revealed that the association was restricted to NSTEMI patients and men with chronic CAD. We have previously shown that other *BTNL2* SNPs, together with HLA-DRB1*01, construct a predisposing ACS haplotype including one SNP in the *BTNL2* promoter region and five downstream SNPs (13). Importantly, the SNPs in the newly identified haplotype are in linkage disequilibrium with these previously reported risk variants, further supporting *BTNL2* as a strong candidate gene for ACS.

NSTEMI and STEMI differ many ways in their clinical characteristics ^30,31^, however, their genetic differences are virtually unexplored. The reason is that the large scale GWAS-studies usually pool all coronary artery disease patients together to gain general genetic patterns in atherosclerosis. The studies cannot distinct different kinds of coronary artery diseases from each other ^32^. A pedantic clinical data gathering is needed for gaining precise data to differentiate NSTEMI from STEMI. We have previously shown that genetic variants on chromosome 1p13.3 are associated with NSTEMI ^33^. It has also been shown that whole blood transcriptome profile discriminates between patients with NSTEMI and STEMI where NSTEMI was consistently associated with T and NK cells and STEMI with antigen processing via MHC class I ^34^. In the present study we showed that *BTNL2* haplotype associates to NSTEMI, but not STEMI further indicating that distinct immune mechanisms underlie the two phenotypes. Another strength of the present study is the high number of *BTNL2* SNPs determined by whole gene sequencing. In general, prior GWA studies have at best included only 10% of the BTNL2 SNPs we identified ^14^. This can explain why the strong association between ACS and BTNL2 has remained largely undetected.

MI is less frequent in women, and they suffer MI usually 10 to 15 years later than men. Ultimate reason for sex differences is unknown; however sex hormones likely explain part of it. We do not yet fully appreciate the underlying reasons for the sex-specific BTNL2 association. We wanted to explore in depth, how BTNL2, cholesterol, and MI are interrelated. Therefore, we conducted a detailed lipoprotein, lipid and metabolite profiling with nuclear magnetic resonance spectroscopy measuring and related it to genetics. The *BTNL2* risk haplotype did not relate to the lipid metabolite levels in case-control comparison when both sexes were compared. However, in disease subgroup analysis, NSTEMI male patients, who are homozygous for the *BTNL2* risk haplotype had an increased concentration of very large HDL particles, when compared to healthy homozygous controls. These HDL particles were lipid-enriched, especially by cholesterol esters. This corroborates a previous study that found the increased HDL diameter and cholesterol content to be associated with CAD risk ^35^. The *BTNL2* gene had been previously associated with larger VLDL particles in the Women’s Genome Health GWA Study consisting of more than 17000 women with cardiovascular disease phenotype ^36^. In our study, the homozygous *BTNL2* risk haplotype was associated with a decrease of the ratio of total and free cholesterol to total lipids in very large VLDL in male NSTEMI patients, while the ratio of triglycerides to total lipids was increased. Similarly, the ratio of free cholesterol to total lipids was decreased in STEMI male patients due to the homozygous *BTNL2* risk haplotype. Furthermore, we found the risk genotype to increase LDL aggregation in a dose-dependent manner, where the risk genotype had highest aggregation susceptibility and wild type samples had the lowest, and those heterozygous for the risk haplotype had an intermediate level of LDL aggregation. Our results corroborate the previous finding of the association between *BTNL2* and very large VLDL metabolism but also point to a functional effect for *BTNL2* and LDL ^36^. Although BTNL2 is not directly involved in lipid metabolism, its immunoregulatory functions may influence inflammatory responses to neutral lipid-enriched lipoproteins which are characteristic of chronic inflammation ^37^.

BTNL2 has immunological properties that are relevant for the pathogenesis of MI. Structurally, BTNL2 is closely related to B7 proteins and has similar immunomodulatory functions. BTNL2 inhibits T cell proliferation and cytokine secretion. It is expressed in lymphoid organs, in activated T cells, B cells and macrophages, and small intestinal epithelial cells ^38,39^. The *BTNL2* gene lies next to other immune response genes in the border between HLA class II and III and several butyrophilingenes (*BTN1A*, *BTN2A* and *BTN3A*) are located in the extended MHC class I region. BTNL and butyrophilin proteins bind phosphoantigens involving intracellular cholesterol metabolism and γδ T cell activation and proliferation^40–43^.

The ligands for BTNL2, like many butyrophilin proteins, are still unidentified. We were, however, able to describe a functional consequence of the risk genotype, whereby while not influencing the size of BTNL2 protein, the risk genotype is associated with increased expression of *BTNL2* in PBMC. Furthermore, in NSTEMI male patients, *BTNL2* risk SNPs are associated with decreased serum concentration of BTNL2. This indicates that in the affected patient group, the NSTEMI males, the upregulated BTNL2 does not enter the circulation in its soluble form. Interestingly, we found that while the disease risk and LDL aggregation increase in the presence of the BTNL2 risk haplotype, the NSTEMI male patients, who are homozygous for the disease associated allele have the lowest risk of cardiovascular death during follow up of nine years. However, those NSTEMI men with the risk haplotype who had died, presented the lowest serum BTNL2 levels. At molecular level, the results raise the possibility that in the NSTEMI male patients carrying the risk genotype, the protein is either not successfully secreted or remains retained membrane-bound on the secreting cells. Either low availability of soluble BTNL2 or high availability of membrane bound BTNL2 may be linked to lower risk of cardiovascular death. Furthermore, in coronary heart disease the previously described protective variant rs28362680 in *BTNL2* is in linkage (D’=1, R^2^=0.311, p <0.0001) with the tagging risk haplotype variant rs2294884 observed in this study, but not with the lead variant rs17202358. Thus, depending on the genomic context, the BTNL2 haplotype may have varying effects and functional consequences. While improved survival is associated with the low-expressing tagging variant rs2294884, when the lead variant rs17202358 is included in the haplotype, even lower levels of serum BTNL2 are linked to poor survival in the patients with both risk alleles.

Among butyrophilin proteins, only BTNL2 has two extracellular immunoglobulin-like domains (IgV and IgC) instead of one, and it lacks the intracellular B30.2 domain, found in other B7 immunoreceptors^44^. Ig domains are involved in a wide variety of functions, which usually require interaction of the intact domain with another protein/molecule. A mutation in such a domain could disturb this interaction. The lead SNP within the risk haplotype, rs17202358, is in total linkage with two nearby *BTNL2* missense variants p.Met380Ile and p.Pro379Leu. Previously p.Met380Ile and p.Pro379Leu have been reported as risk SNPs for rheumatoid arthritis, osteoarthritis of the knee and sarcoidosis, but not for CAD^45–47^. The change to mutant variant in these positions is predicted to disturb the local structure by loss of an exposed methionine sidechain at position 380 and/or a loss of a disulphide bridge between cysteine at position 381 and an adjoining cysteine, thereby inhibiting receptor interactions. The total LD between the lead SNP of the risk haplotype and previously described disease-associated missense variants offers a plausible link between the discovered haplotype’s disease association and altered protein functionality. The association between p.Met380Ile and p.Pro379Leu and CAD should be subjected to sub-analysis accounting for sex and NSTEMI/STEMI diagnoses of patients.

Lack of previous CAD GWAS associations with *BTNL2* highlights the complexity of the MHC region and warrants a targeted analytic approach to decipher the immunogenetic risk factors for complex diseases such as MI. We report a sex bias in CAD patients in relation to immunogenetic risk factors implicating the significance of the immune system for differential manifestation of CAD and especially for chronic disease. Recently, other MHC genes such as *HLA* and complement *C4* were associated with sexual dimorphism in T cell selection and expansion and in vulnerability to diverse diseases ^48,49^.

The study has limitations. The study populations consist only Finnish patients from one centre, who were compared against Finnish controls. However, the gathered cohorts are large and comprise different kind on patients with coronary artery disease. The cohort used in this study was a prospective cohort consisting of patients who survived to hospital admission and approximately 25% of the myocardial infarction patients die outside hospital. We cannot rule out the effect of a potential selection bias’s effect on the better survival in risk haplotype positive males who were predisposed to NSTEMI. However, allele frequency data does not support this scenario, because the GG risk haplotype was less frequent in controls than in cases.

In a unique genetic study of Finnish MI patients and controls, we have replicated our previous finding of a predisposing immunogenetic mechanism that contributes to the high risk for cardiovascular disease observed in this population. Specifically, we have shown that the discovered *BTNL2* risk haplotype in NSTEMI males is linked to i) increased expression of BTNL2 in PBMC, ii) decreased BTNL2 protein concentration in serum, iii) increased concentration of very large HDL particles, iv) cholesterol ester enrichment of very large VLDL, v) increased aggregation of LDL, and vi) improved long-term survival of the patients. The results indicate the diverse effects of BTNL2 on the disease highlighting the importance of sex specific diagnostic precision in studies exploring MI disease mechanisms.

## Supplementary Material

Supplementary Files

This is a list of supplementary files associated with this preprint. Click to download.

• TABLES.docx

• supp.docx

## Data Availability

Qualified researchers can request access to additional data from the corresponding author.
